# Wind Speed Interval Prediction Based on Bayesian Optimized Spatio-Temporal Integration and Compression Deep Residual Network

**DOI:** 10.3390/s25206370

**Published:** 2025-10-15

**Authors:** Yun Wu, Yongzhen Gong, Xiaoguo Chen, Xingang Wang, Xiaoyong Li

**Affiliations:** 1 Guangdong Provincial Key Laboratory of Petrochemical Equipment Fault Diagnosis, School of Mechanical and Electrical Engineering, Guangdong University of Petrochemical Technology, Maoming 525000, China; 2 College of Electrical and Information Engineering, Hunan University, Changsha 410082, China; 3 School of Economics and Management, Guangdong University of Petrochemical Technology, Maoming 525000, China

**Keywords:** wind speed prediction, temporal convolutional networks, NKDE interval prediction, residual structure, Bayesian optimization

## Abstract

To address the challenge of high wind speed variability in wind farm planning, a small-sample-based spatio-temporal fusion and compression deep residual point prediction model, STiCDRS (Spatio-Temporal integration and Compression Deep Residual), is proposed. This model is designed to deeply explore the spatial and temporal characteristics within wind speed sequences to enhance the accuracy of point predictions. Initially, the spatio-temporal integration and compression deep residual network is employed to obtain point prediction results. Subsequently, an innovative hybrid model, STiCDRS-NKDE (STiCDRS-Nonparametric Kernel Density Estimation), is introduced to achieve interval predictions, thereby providing more reliable probabilistic forecasts of wind speed. The hyper-parameters of the model are optimized using Bayesian optimization, ensuring efficient and automated tuning. Finally, a case study involving wind speed forecasting at a wind farm in Inner Mongolia, China, is conducted, comparing the performance of the STiCDRS model with traditional models. Experimental results demonstrate that in comparison to other models, the proposed STiCDRS-NKDE model delivers superior point prediction accuracy, appropriate interval predictions, and reliable probabilistic forecasting outcomes, fully showcasing its significant potential in the domain of wind speed forecasting.

## 1. Introduction

As the global demand for renewable energy grows, wind energy has become an important option for replacing traditional energy sources due to its renewable and clean nature [1]. Wind power generation technology uses wind energy to convert into electricity, and its power generation is closely related to wind speed. Because of this, accurate prediction of wind speed is particularly important. It is of great practical significance to optimize grid scheduling and improve the accurate assessment of wind farm capacity through effective prediction of wind speed, which in turn provides an important basis for determining the price of wind power feed-in tariffs [2]. However, the volatility and randomness of wind energy bring great challenges to wind speed prediction.

Traditional wind speed prediction methods mainly rely on physically driven models [3] or statistical methods [4], which are effective in dealing with large-scale or complex data, but face the following problems: (1) difficulty in model training: it is difficult to learn the complex and volatile patterns of wind speed from limited data; (2) risk of overfitting [5]: over-adaptation to random fluctuations or noises in the training-limited data leads to significant performance differences in different datasets with large differences in performance; (3) increased prediction uncertainty: due to the limitation of the data volume, the model’s prediction of wind speed volatility is often accompanied by a high level of uncertainty.

Compared with traditional statistical methods, machine learning methods can deal with complex nonlinear relationships more effectively by mining the intrinsic connection of data through feature learning [6], thus improving the accuracy and adaptability of prediction. Commonly used machine learning methods include Gaussian process regression (GPR) [7], K-nearest neighbors (KNNs) [8], random forest regressor (RFR) [9], and support vector regression (SVR) [10,11]. Compared to these shallow learning models, deep learning models adopt a distributed, hierarchical feature representation that automatically extracts intrinsic abstract features and hidden invariant structures from the lowest to the highest level of data [11]. This excellent feature extraction capability has brought deep learning models to the attention of scholars in the field of wind speed and wind power prediction. In the field of wind power, the temporal convolutional network (TCN) [12] is very widely used, providing a more efficient and stable framework for dealing with temporal prediction tasks [13]. The TCN has been progressively applied in the fields of electric load forecasting, wind speed forecasting, and photovoltaic forecasting. For example, the literature [14] proposed a short-term wind speed prediction method based on temporal convolutional networks, and the comparison results show that the model has the highest prediction accuracy and the best prediction results.

Although the TCN performs well in time-series data processing, it has some shortcomings: (1) in small sample scenarios, this deep learning automatic feature extraction capability is limited, and its serialized data processing characteristic leads to computational inefficiency, which in turn increases the complexity of parameter optimization and model application; (2) its performance suffers when dealing with sequences with data redundancy or more noise, and the model takes these irrelevant information into account in the final prediction, leading to a decrease in prediction accuracy; (3) the setting of model hyper-parameters relies on the experience from previous studies or practices to be simply tried and tested, which is poorly adaptive and inefficient, which is a great challenge for dealing with sequential data with complex temporal dynamics.

In addition, traditional wind speed forecasting studies mainly focus on point forecasting, i.e., predicting wind speed values at a specific moment in the future. One of the biggest problems facing point forecasting for small sample datasets is high uncertainty. Given the volatility of wind speeds and the variability of the environment, along with the limited number of data points, this leads to a decrease in the credibility of the prediction results. Point forecasts are often insufficient to provide comprehensive decision support, limiting the scope of their application in energy system planning and operation. To overcome these limitations, interval forecasting has emerged. Interval forecasting provides a way to quantify the uncertainty of future wind speeds and enhance the reliability of forecasts by predicting an interval that may contain future wind speed values instead of a single value [15]. Commonly used interval prediction methods include bootstrap [16], linear regression confidence intervals [17], Gaussian process regression [18], and auto-regressive moving averages (ARMA) models [19] and their variants.

To address the above bottlenecks, a wind speed interval prediction model, STiCDRS-NKDE, based on improved TCN is proposed for the first time in this paper. Firstly, an innovative end-to-end model is proposed for wind speed prediction, in which the 1 × 1 convolutional layer in the traditional residual structure of the TCN is replaced by shared weight minimal gated memory (MGM) [20]. Secondly, a soft threshold residual structure (deep threshold residual structure) based on attention mechanism is introduced after the improved TCN feature output layer [21]. Thirdly, a soft residual shrinkage network (DRSN) based on the attention mechanism is introduced after the improved TCN feature output layer; furthermore, this paper adopts a Bayesian optimization approach to achieve efficient and automated tuning of the model hyper-parameters. Finally, on the basis of improving the STiCDRS point prediction results, the interval prediction of the model output is achieved by nonparametric kernel density estimation. The results show that the proposed model STiCDRS-NKDE for wind speed interval prediction achieves a significant improvement in wind speed prediction accuracy.

## 2. Improved STiCDRS Wind Speed Point Prediction Model Based on Bayesian Optimization

### 2.1. Temporal Convolutional Network

**Figure 1:**
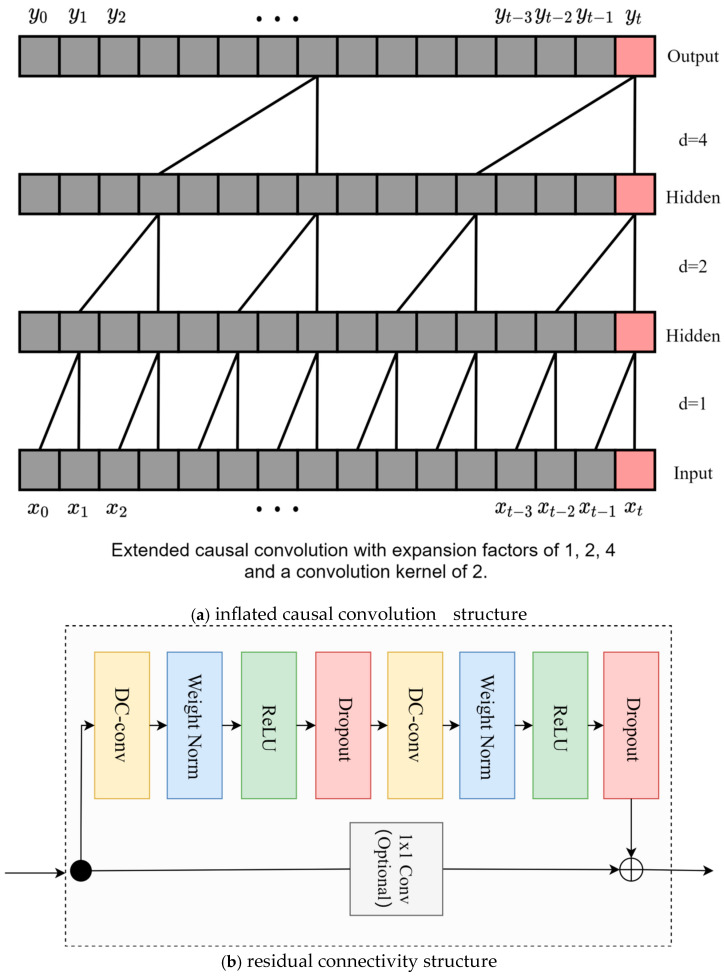
Schematic diagram of the traditional TCN structure.

The TCN is a neural network architecture designed using inflated causal convolution. The construction of the subsequent point prediction model will be based on the TCN model for structural reconfiguration, which can significantly reduce the number of parameters of the model, and the implementation of its most core structure includes the inflated causal convolution and residual connectivity structure, as shown in Figure 1a,b.

In the case of a one-dimensional time series, the causal convolution is computed as follows:

(1) $$y_{t}=\sum_{i=1}^{k}\omega_{i}\cdot x_{t-i+1}$$

where $y_{t}$ is the *t*-th element of the output sequence, $x_{t}$ is the *t*-th element of the input sequence x, $\omega_{i}$ is the *i*-th weight of the convolution kernel, and *k* is the size of the convolution kernel.

Expansion convolution expands the receptive field of a convolution kernel by introducing spacing in the convolution kernel without increasing the size of the kernel. Given an input sequence *x*, a convolution kernel ω of size *k*, and an expansion rate *d*, the computation of the expansion convolution is denoted as follows:

(2) $$y_{t}=\sum_{i=1}^{k}\omega_{i}\cdot x_{t+(i-1)\cdot d}$$

where *d* is the expansion rate.

A residual block contains two inflated causal convolutional layers, each of which also adds WeightNorm and Dropout to regularize the network, in addition to a 1 × 1 convolution to keep the inputs and outputs of the same scale, as shown in Figure 1b. Specifically, for a basic residual block with input sequence x and output $y_{out}$, the residual join is computed as follows:

(3) $$y_{out}=F(x)+C_{opt}(x)$$

where *x* is the original input, F(x) is the nonlinear transformation of the backbone layer in the residual block, and $C_{opt}(x)$ is the transformation of the residual structure (which can be divided into TCN residual structure, where the output is transformed by 1 × 1 convolution, and traditional residual structure, where the output is *x*). The final output is the concatenation of the original input and the result vector after nonlinear transformation.

### 2.2. Deep Residual Shrinkage Network

The DRSN is an improved residual structure. To further improve the prediction accuracy, the DRSN will be used to further improve the residual module of TCN, which further enhances the feature extraction and representation capability of the model by combining the residual linkage in the TCN and the soft-threshold residuals based on the attention mechanism for combined filtering.

**Figure 2:**
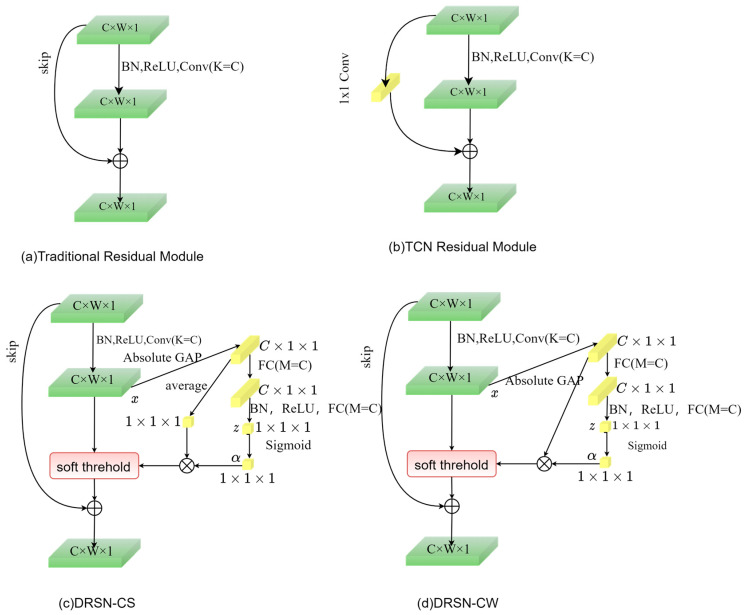
Structural diagrams of the TCN residual module and the DRSN-CS module in comparison with the DRSN-CW module.

The traditional residual structure is shown in Figure 2a,b, while Figure 2c,d show two variants of the DRSN, i.e., deep residual shared shrinkage network with channel-shared thresholds (DRSN-CS) and deep residual point-by-point shrinkage network (deep residual shrinkage network with channel-wise thresholds, DRSN-CW). Compared with the DRSN-CS module, the threshold obtained by the DRSN-CW residual module is a vector, i.e., each channel corresponds to a shrinkage threshold, so the DRSN method adopted in this paper defaults to DRSN-CW. In contrast to the traditional residual module, the DRSN adds a soft threshold and attention mechanism to the traditional structure.

The primary purpose of the soft threshold is to eliminate features with absolute values below a specified threshold, effectively transforming values near zero into zero. This function compresses the input data towards zero. The soft threshold function is defined as follows:

(4) $$y=\left\{\begin{array}{l} x-\tau \;x>\tau \\ \;0\;-\tau \leq x\leq \tau \\ x+\tau \;x<-\tau \end{array}\right.$$

where *x* is the input feature, *y* is the output feature, and τ is the threshold.

Squeeze-and-excitation network (SENet), as the main part of the attention mechanism, enables the model to automatically learn a set of weighting coefficients, weight the learned weights to each channel in the feature map, evaluate the importance of each channel, and thus enable the model to distinguish between important and redundant feature channels. In SENet, the learning path passes through the global pool, the fully connected layer, the ReLU activation function, and ends with the Sigmoid function.

In deep residual shrinkage networks, the SE module automatically learns thresholds and combines them with soft threshold denoising, which solves the problem of threshold selection.

### 2.3. Bayesian Optimization

**Figure 3:**
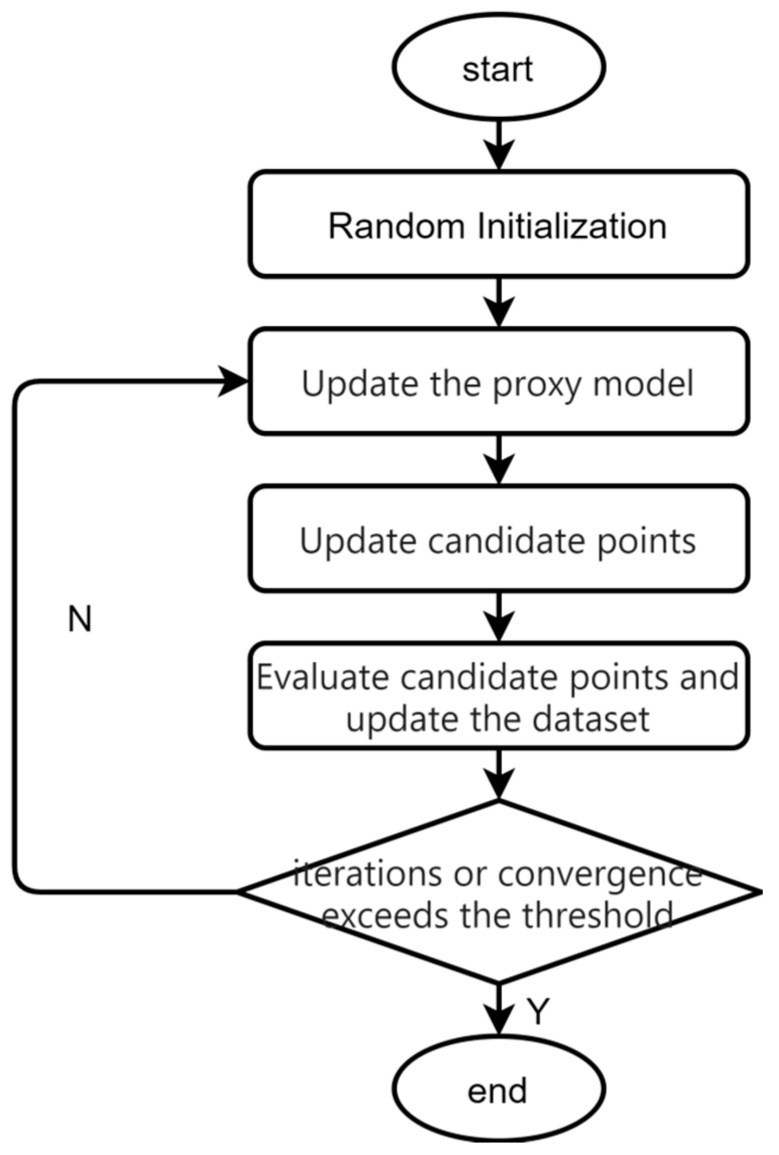
Flowchart of Bayesian optimization hyper-parameters.

Bayesian optimization is a commonly used black-box function optimization method [22]. It can effectively solve the problem that the hyper-parameters of the model in this paper rely on previous experience to simply try to put together and have poor adaptability, and achieve efficient and automated tuning of the hyper-parameters. The Bayesian optimization flowchart is shown in Figure 3.

The Gaussian process model assumes that the value of the objective function at any point in the parameter space obeys a Gaussian distribution:

(5) $$f(x)\sim N(\mu (x),k(x,x^{\prime }))$$

where μ(x) is the mean function and $k(x,x^{\prime })$ is the covariance function.

After observing some sample points of the objective function, the parameters of the model are updated using Bayes’ theorem based on the available observations $D={\left\{(x_{i},y_{i})\right\}}_{i=1}^{n}$. The posterior probability distribution of the parameters is obtained as follows:

(6) p(f(x)|D)∝p(y|x,D)p(f(x)|D)

where *d* is the likelihood function of the observed data and *p* is the prior posterior distribution.

The a posteriori probability represents the uncertainty and distribution of parameters given the observed data. The sampling strategy identifies the next most likely extreme point based on this posterior probability distribution. The Bayesian optimization algorithm iteratively optimizes the objective function by updating the a posteriori distribution and selecting subsequent parameter points for evaluation until a predefined termination condition is met.

### 2.4. Point Prediction Model Construction

In the traditional residual structure of TCN, there are some defects in the 1 × 1 convolutional layer. The 1 × 1 convolutional layer only adjusts the number of channels and has no explicit memory capability, which may lead to the loss of some important time-series information during the propagation process. The sensory field of the 1 × 1 convolutional layer is very small, and it can only capture the local information. It is relatively weak for the modeling of long-term dependencies, and the local sensory capability is limited. Therefore, this paper innovatively proposes the use of MGM to replace the 1 × 1 convolutional network to obtain the new network STiC.

**Figure 4:**
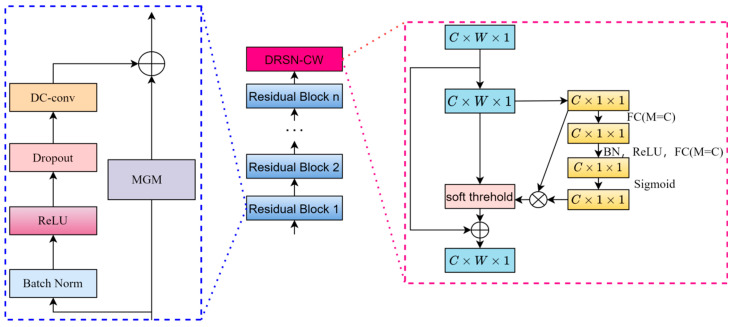
Model structure diagram of the STiCDR.

However, the STiC structure still has some limitations: it is difficult to deal with noisy and redundant feature information, and the features of wind speed data itself cannot be filtered for importance. To address these two shortcomings, this paper optimizes the preliminary improved STiC residual module even further: the DRSN module is added after the feature output layer of the improved STiC, and an improved deep learning model based on spatio-temporal feature fusion and residual attention soft-thresholding mechanism, named STiCDRS in this paper, is proposed for the point prediction of wind speeds. Its structure is shown in Figure 4.

**Figure 5:**
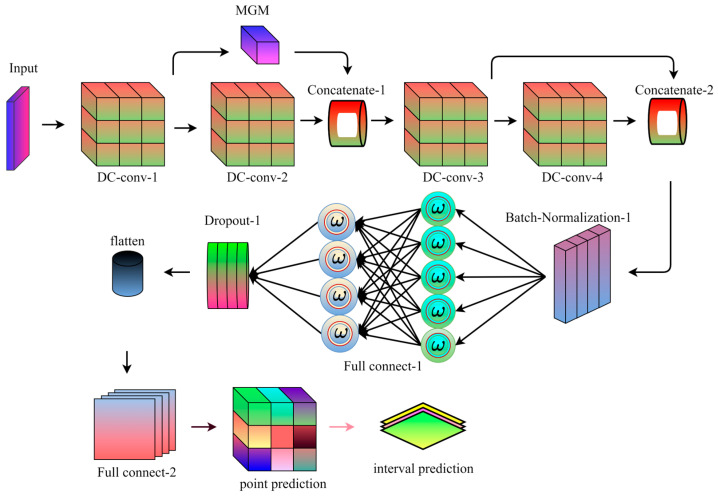
STiCDRS point prediction flowchart.

For the proposed point prediction model, the prediction process is shown in Figure 5.

The specific process is as follows: the model receives the input data, the data passes through the first depthwise convolutional layer (DC-conv-1), and then the data passes through the second depthwise convolutional layer (DC-conv-2) and the first shared weight coefficients minimal gated memory (MGM) at the same time to further extract the high-level features. The outputs of DC-conv-1 and MGM are stitched together with the DC-conv-2 output, and the spliced data flow through the third depthwise convolutional layer (DC-conv-3) to further process the features. The data are sequentially passed through skip module and the fourth depthwise convolutional layer (DC-conv-4) to enhance the model’s ability to process the time-series data and to extract deeper features. The outputs of the outputs of skip module and DC-conv-4 are spliced to further fuse the feature information. The spliced data are processed through batch normalization to speed up training and improve model stability. The data stream passes through the fully connected layer, which begins to convert the features into the format required for prediction. The data is flattened into one dimension after a discard layer to reduce overfitting before being passed to the next fully connected layer. The data then passes through another fully connected layer for the final prediction task, resulting in the final output point prediction.

## 3. Improved STiCDRS-NKDE Wind Speed Interval Prediction Model Based on Bayesian Optimization

### 3.1. NKDE Interval Prediction

The NKDE algorithm is a non-parametric test method that does not require prior knowledge of data distribution and does not make any assumptions about data distribution. It can obtain the distribution of the corresponding predicted period from the data sample itself. Since it can directly estimate the density of random variables without assuming the superiority of the distribution, it is suitable for wind speed interval prediction research. The expression of the NKDE algorithm is usually the same as that of KDE, specifically

(7) $$\hat{f}(x)=\frac{1}{nh}\sum_{i=1}^{n}K(\frac{x-X_{i}}{h})$$

where *N* is the total number of samples, and h>0 indicates the window width, also known as the smoothing parameter, which can be set arbitrarily. In this research, h was calculated according to Silverman’s Rule of Thumb [23]; *x* is the random variable of the wind speed point prediction error; *X_i_* is the *i*-th sample point value of the wind speed point prediction error; and K(⋅) represents the selected non-negative kernel function. Using different kernel functions will produce different estimation results. Usually, the Gaussian kernel function is used.

### 3.2. Interval Prediction Model Construction

The specific principle of combining deep learning with NKDE for interval prediction is to first use the deep learning model to obtain the corresponding point prediction wind speed value and prediction error set, then divide the error set into individual sub-segments, and then use the NKDE algorithm to construct a probability interval prediction model after interpolation for each sub-segment to obtain a probability density curve. Finally, the prediction result values of each sub-segment are reorganized to obtain the probability prediction interval of the wind speed. The specific principle of combining deep learning with NKDE for interval prediction is to use the parameters and kernel function in the NKDE model as the input layer and hidden layer of deep learning, respectively [24], and use the nonlinear characteristics of deep learning to improve the prediction accuracy and generalization ability of the model. At the same time, the deep learning model can also automatically discover and extract hidden features in wind speed data information by learning a large amount of data, thereby improving interval prediction performance while meeting the requirements of accuracy and confidence [25].

**Figure 6:**
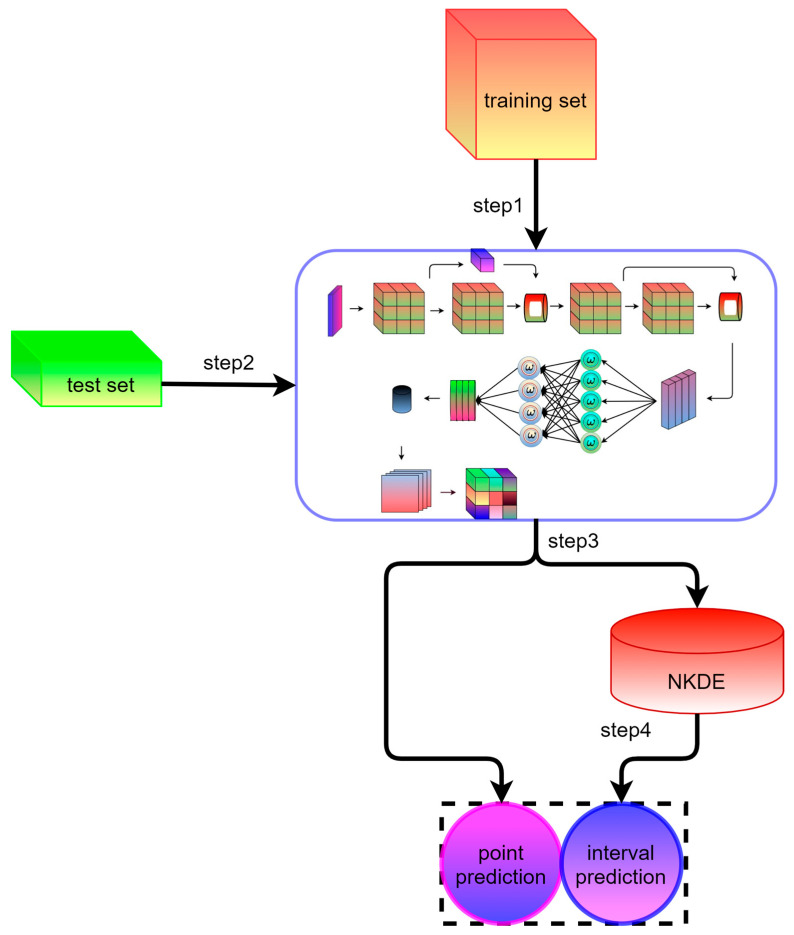
Schematic diagram of STiCDRS-NKDE training and testing process.

Therefore, an idea to obtain high-precision point prediction, high-reliability interval prediction, and probabilistic prediction is to propose the reliable probabilistic prediction performance of STiCDRS fused with NKDE by combining the advantages of STiCDRS point prediction, i.e., the wind speed interval prediction method of STiCDRS-NKDE. The training and testing process of the STiCDRS-NKDE model is shown in Figure 6.

The specific steps of the method are as follows:

Step 1: Complete the training of the STiCDRS model in the single point prediction process;

Step 2: Feeding the test set into the STiCDRS model;

Step 3: Model prediction and thus obtaining a single point prediction value;

Step 4: Inputting the single-point prediction value into the NKDE model to obtain the interval prediction value.

**Figure 7:**
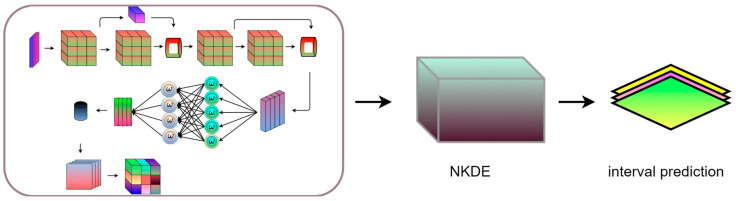
Structural diagram of the STiCDRS-NKDE model.

The interval prediction model inherits the high accuracy of the STiCDRS model in single-point prediction, which makes it possible to obtain a more reliable interval prediction range and probability density function (PDF) when constructing the NKDE model between the point prediction value and the observation value, thus obtaining the final STiCDRS-NKDE model, as shown in Figure 7.

### 3.3. Evaluation Metrics

In order to fully verify the effectiveness of the proposed interval prediction model, in which point prediction is the basis of interval prediction, this study uses three point evaluation indexes [26], three interval evaluation indexes, and one computational efficiency evaluation index to evaluate the performance of the model. It uses the same evaluation indexes to compare the interval prediction model of this paper with the classical model, which has a similar structure. The point prediction evaluation method mainly involves root mean square error (*RMSE*), mean absolute error (*MAE*), and coefficient of determination (*R*^2^). Let the predicted target value be *x* and the real target value at the corresponding moment be y. Then, the evaluation method is calculated as follows:

(8) $$MAE=\frac{1}{N}\sum_{i=1}^{N}\left|\left(h(x_{i})-y_{i}\right)\right|$$

(9) $$RMSE=\sqrt{\frac{1}{N}\sum_{i=1}^{N}({\left(h(x_{i})-y_{i}\right)}^{2}}$$

(10) $$R^{2}=1-\frac{\sum_{i=1}^{N}({\left(h(x_{i})-y_{i}\right)}^{2}}{\sum_{i=1}^{N}({\left(y_{i}-\bar{y}\right)}^{2}}$$

where *N* is the number of test samples, $\bar{y}$ is the mean value of the target value, $y_{i}$ is the predicted value, and $h(x_{i})$ is the predicted value.

The smaller values of MAE and RMSE indicate that the point prediction performance of the model is better, while R takes values ranging from −1 to 1. When the value of R is close to −1 or 1, it indicates that there is a strong linear relationship between the two variables, and when it is close to 0, it indicates that the linear relationship is weak or non-existent.

The interval prediction performance evaluation metrics used in this study include coverage probability (*CP*), comprehensive evaluation metrics, and probability integral transform (*PIT*) involving probabilistic prediction [27].

Coverage CP is defined as the probability that an observation falls within a prediction interval at α confidence level. It is calculated as shown in Equation (11):

(11) $$CP=\frac{C_{\alpha }}{T_{e}}$$

where $C_{\alpha }$ is the number of samples whose observations fall within the prediction interval, and $T_{e}$ is the total number of samples in the test set.

Assuming that the interval is wide enough, it could easily lead to a value of 100 per cent for CP. Such intervals do not provide valid information about the uncertainty of the prediction, which makes the prediction interval of no practical value. The interval width percentage (MEAN WIDTH PERCENTAGE) MWP is defined as the average percentage of the interval width over the observations to ensure the validity of the interval and is given in Equation (12) [28]:

(12) $$MWP=\frac{1}{T_{e}}\sum_{i=1}^{T_{e}}\frac{h_{\max_{i}}-h_{\min_{i}}}{h(x_{i})}$$

where $h_{\max_{i}}$ and $h_{\min_{i}}$ are the upper and lower limits of the prediction interval for the *i*-th sample point, and c is the observed value for the *i*-th sample point.

The more ideal interval prediction result should have both a larger value of CP and a smaller value of MWP. Therefore, it is considered by defining the combined evaluation index metric MC of the two, which is calculated as shown in (13):

(13) $$MC=\frac{MWP}{CP}$$

By combining CP and MWP, it can be concluded that the prediction performance of the interval indicated is higher when MC is smaller.

Reliability refers to the statistical consistency of predictions and observations, and the uniform probability plot of PIT values is used to assess the prediction reliability, which is calculated as shown in (14) [29]:

(14) $$PIT=\int_{-\infty }^{h(x_{i})}p(t)dt$$

Here, $h(x_{i})$ denotes the corresponding point prediction, where PIT can usually be shown by a P-P plot, i.e., plotting the *CDF* of the *PIT* against the *CDF* of a standard uniform distribution. The P-P plot plotted is closer to a straight line when the prediction is more accurate.

Average training time (*ATT*) is the average time in seconds for the model to be trained five times on the training set. It is used to measure the training efficiency of the model, and the model with a shorter training time has higher utility and efficiency in dealing with large-scale data sets.

The calculation formula is as follows:

(15) $$ATT=\frac{1}{5}\sum_{i=1}^{5}T_{i}$$

where $T_{i}$ is the time of the *i*-th training.

## 4. Experiments and Results

### 4.1. Description of the Experimental Dataset

**Figure 8:**
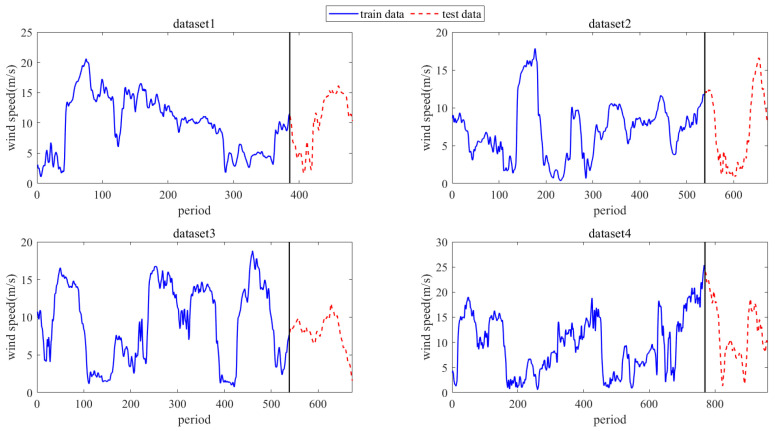
Schematic diagram for dividing the training and testing sets of 4 wind speed dataset waveform atlases.

To assess the effectiveness of the proposed method STiCDRS in this paper, this experiment studies four datasets from the wind farm in Inner Mongolia, China, from 20 March 2016 to 19 May 2016, each with a wind speed data step of 15 min [30], which corresponds to datasets 1, 2, 3, and 4, respectively. The first 8 cycle points are used as inputs to predict the current point. The training and testing set division of the dataset is shown in Figure 8.

Dataset 1 runs from 23 April 2016 (00:00) to 28 April 2016 (07:00), with a sampling period of 15 min, and contains 481 cycle points. Dividing the training and test sets according to the ratio of 8:2, the first 385 cycle points are taken as the training set and the last 96 cycle points are taken as the test set. Dataset 2 runs from 20 March 2016 (00:00) to 27 March 2016 (00:00), with a sampling period of 15 min, and contains a total of 673 cycle points, dividing the training and testing sets in accordance with the ratio of 8:2, taking the first 538 cycle points as the training set and the last 135 cycle points as the testing set. Dataset 3 runs from 11 April 2016 (00:00) to 18 April 2016 (00:00), with a sampling period of 15 min, containing 673 cycle points, dividing the training and testing sets according to a ratio of 8:2, taking the first 538 cycle points as the training set and the last 135 cycle points as the testing set. Dataset 4 is from 07:00, 3 May 2016, to 07:00, 13 May 2016, with a sampling period of 15 min, containing a total of 961 cycle points, dividing the training and testing sets according to the ratio of 8:2, taking the first 769 cycle points as the training set and the last 162 cycle points as the testing set.

### 4.2. Parameter Configuration

For the parameter settings of the model during training, for some hyper-parameters that are not easy to select using optimization methods, the commonly used parameter settings for deep learning model training are referred to. The Kaiming method is used to initialize the weight coefficients of the CNN layer and the fully connected layer [31], and the orthogonal method is used to initialize the weight coefficients of the MGM layer and the LSTM layer, which is conducive to accelerating the convergence of the model. During the network training process, the Adam optimizer is used to make the model converge faster and more robust, while reducing the verification set test. The processed dataset is randomly shuffled and divided into training and test sets with an 8:2 ratio. The specific settings are shown in Table 1.

For convolutional kernel size, the number of convolutional kernels, hidden unit size of the fully connected layer, batch size, learning rate, and regularization coefficients are subsequently combined for adaptive selection using Bayesian optimization methods.

### 4.3. Model Hyper-Parameter Selection Experiments

In machine learning and deep learning, the performance of a model is usually affected by hyper-parameters, so choosing the right combination of hyper-parameters is crucial to the performance of the model. A commonly used approach is to adaptively select hyper-parameter combinations via a Bayesian optimization algorithm. The Bayesian optimization algorithm allows the algorithm to adaptively select hyper-parameter combinations that are more likely to reach the optimal value by continuously trying different hyper-parameter combinations and updating the probabilistic model of the hyper-parameter combinations based on the output of the objective function. This method can be more efficient than the traditional grid search or random search to search for optimal hyper-parameter combinations. In addition, in order to fully illustrate the effectiveness of the proposed STiCDRS in wind speed prediction, this study first optimizes the combination of key parameters (shown in Table 2) of TCN models with similar structures through Bayesian optimization to find the optimal hyper-parameter combinations, and then the other models for comparison and STiCDRS are set to have the same hyper-parameters. The optimal hyper-parameter combinations of the TCN models are shown in Table 2.

Set the range of convolutional kernel size optimization in the TCN model network as [1, 4], the range of convolutional kernel number optimization as [8, 256], the range of fully connected layer hidden unit size optimization as [64, 512], the range of batch size optimization as [8, 16, 32, 64, 128, 256], the range of learning rate optimization as [0.001, 0.01], and the range of regularization coefficient optimization as [0.1, 0.9]. Based on Bayesian optimization for hyper-parameter optimization of the TCN model, calculate the minimum value in the returned objective function and record the hyper-parameter combination that produces the minimum loss to obtain the final hyper-parameter combination table.

**Figure 9:**
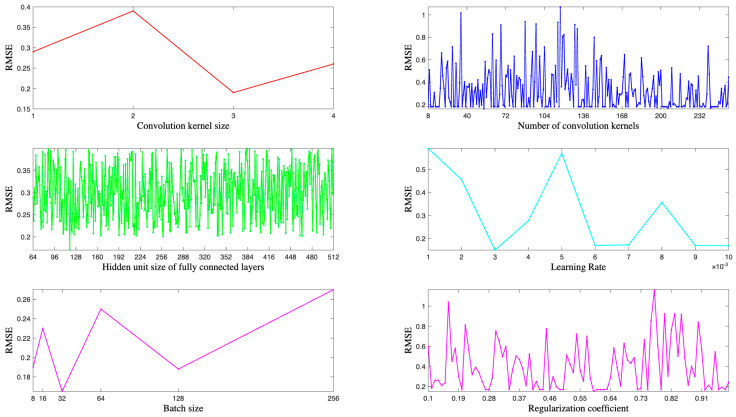
The loss function value of the benchmark model with different hyper-parameters in the Bayesian optimization process.

Specifically, Figure 9 shows the loss function values of the baseline model when optimizing individual hyper-parameters during Bayesian optimization. It is clear that the loss function value is minimized when the convolution kernel size is 3, the convolution kernel size is 31, the learning rate is 0.003, and the L2 regularization coefficient is 0.93. Although the minimum loss value corresponding to the number of hidden units in the fully connected layer is not obvious, a comparison of specific values shows that the loss function value performs best when the number of hidden units is 119.

The output parameter dimensions and sizes corresponding to the structural layers of the DRTS-Net framework are shown in Table 3.

### 4.4. Comparative Analysis of Experimental Results

**Figure 10:**
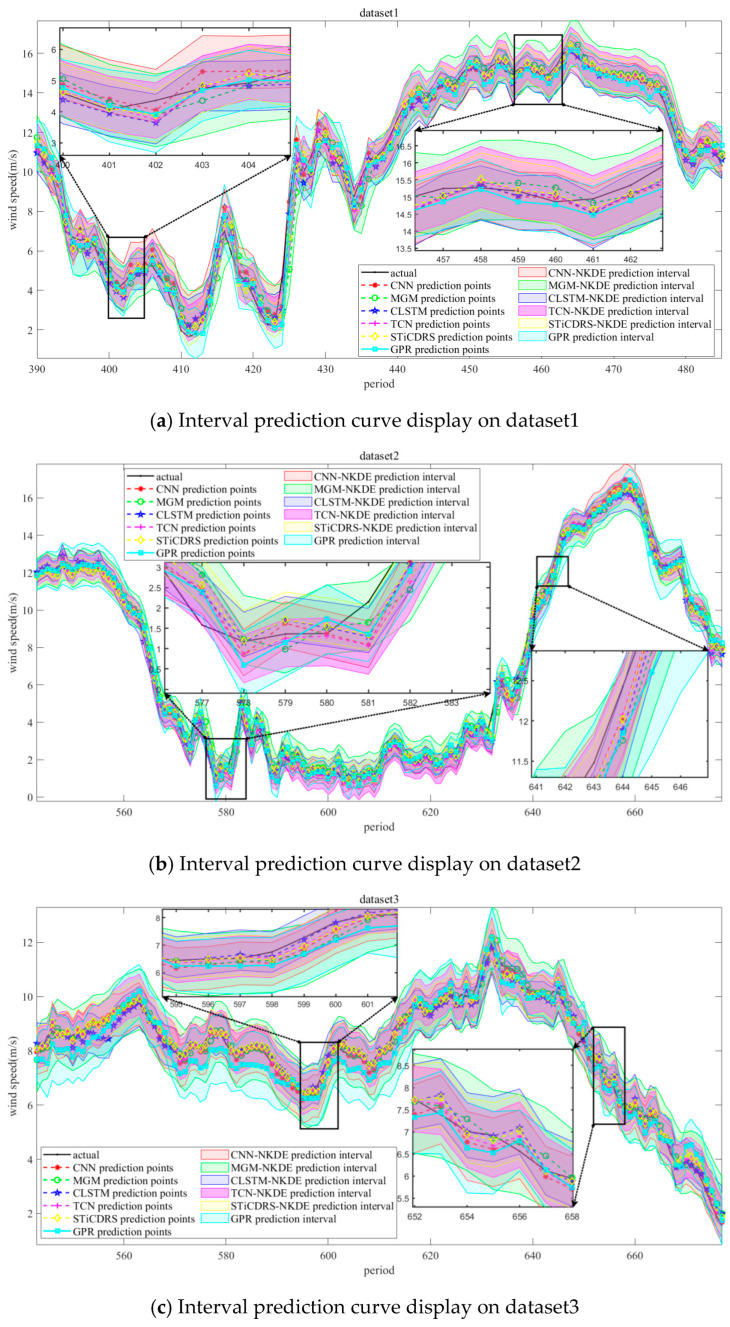

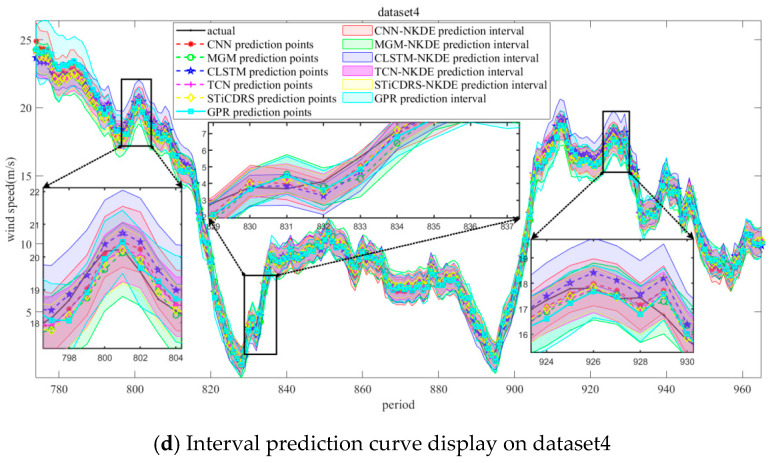
All comparison model predictions correspond to the point prediction and interval prediction results of the 4 test datasets.

Simulation experiments are carried out on four wind speed datasets selected for this study through the parameter settings described above, in which the predicted and observed values and prediction intervals obtained from the CNN, MGM, CLSTM, TCN, STiCDRS, and GPR models on the test set are shown in Figure 10.

**Figure 11:**
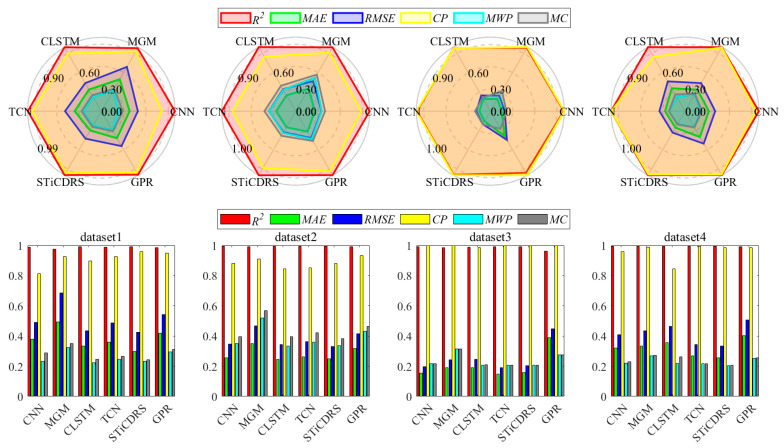
Radar and bar charts of evaluation metric values for all model point predictions and interval predictions corresponding to the four datasets.

The radar and bar charts in Figure 11 illustrate the evaluation metrics for all model point and interval prediction results across four datasets. The radar charts clearly show that the STiCDRS model outperforms the other models in six key metrics. Notably, for the forward metrics *R*^2^ and CP, the STiCDRS model achieves the largest radius, nearing the optimal value of 0.99, indicating its superior prediction capability.

In terms of reverse metrics, the STiCDRS indicators are closest to the center of the circle, with most values falling below 0.3, further highlighting its exceptional predictive performance.

The bar chart reinforces these findings, with the red and yellow bars representing the two positive metrics. Here, the STiCDRS model consistently approaches a value of 1 across all datasets, outperforming all other models. Meanwhile, the green, dark blue, light blue, and gray bars denote the four negative metrics, where the STiCDRS model maintains values lower than 0.4 in all datasets, demonstrating its overall superiority.

Importantly, the STiCDRS model shows remarkable consistency, with nearly all metrics below 0.4, which is significantly better than competing models. In particular, for dataset 3, the STiCDRS model achieves a value close to 0.2, which is notably lower than that of several commonly used models, underscoring its effectiveness in predictive accuracy.

The results of the specific evaluation metrics for the six models on the test set on the four datasets are shown in Table 4, with the optimal results highlighted in red, the sub-optimal results highlighted in purple, and the worst results highlighted in green (10 runs were run, with the average being the final result). Among them, the point prediction evaluation metrics *R*, *MAE*, and *RMSE* and the interval prediction evaluation metrics *CP*, *MWP*, and *MC* using the STiCDRS-NKDE model have the best combined results among all the models, which is a significant improvement compared to the TCNs without spatial fusion of the STiCDRS structure. Comparison shows that the models with a spatial feature extraction module, such as CNN, CLSTM, TCN, and STiCDRS, outperform the MGM model using only the temporal extraction module in the prediction of wind speed dataset. Meanwhile, the STiCDRS model with the fused spatio-temporal residual module outperforms the other basic residual structure models to further improve the model’s performance, which suggests that the improved fusion of the spatio-temporal features within the residual module can improve the accuracy of the models without significantly increasing the training time of the deep learning models. All models are tested with the same datasets and initialized under the same random weights during the test, so on the whole, (1) the coupling of the models improves the ability to map between the variables; (2) the residual structure of null-time fusion improves the models; and (3) the fusion of the soft-threshold residuals based on the attentional mechanism further improves the model’s accuracy. The results all show that the model will have more accurate prediction ability.

**Figure 12:**
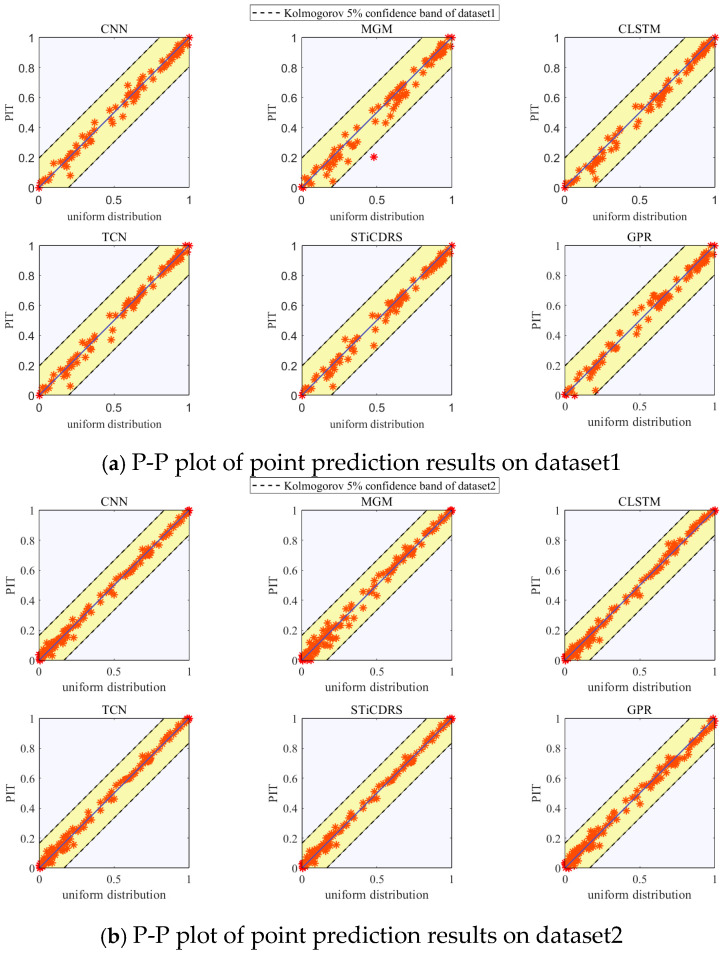

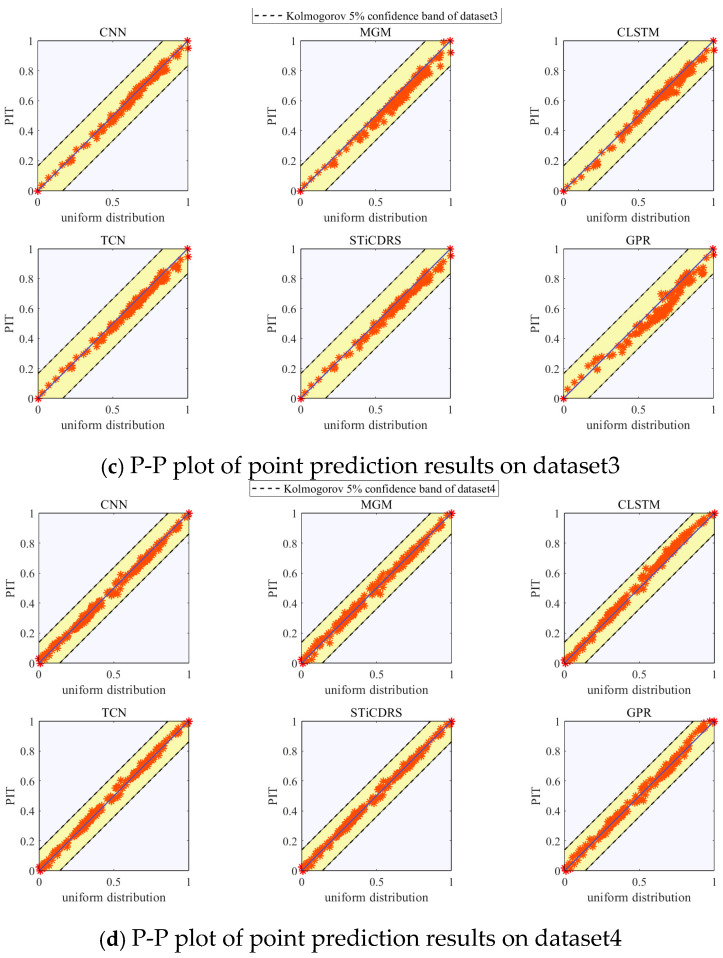
P-P plot of all model point prediction results corresponding to 4 datasets.

The P-P plots showing the *PIT* presentation results of the test set corresponding to the CNN, MGM, CLSTM, TCN, STiCDRS, and GPR models’ point prediction are shown in Figure 12. The P-P plots of the point prediction results obtained by all the models show a uniform distribution on both sides of the diagonal line and are uniformly covered in the range [0, 1], which indicates that the point prediction PIT results obey a uniform distribution, and at the same time, it shows that the prediction results are all more reliable. Meanwhile, the PIT points of the point prediction results of all models in this study for the test set of four datasets are distributed within Kolmogorov’s 5% significance range, indicating that the prediction results’ PDF are highly reliable. The CNN-NKDE, MGM-NKDE, CLSTM-NKDE, TCN-NKDE, STiCDRS-NKDE, and GPR models show good reliability in the probabilistic prediction of wind speed prediction, in which the results of the STiCDRS-NKDE model can be better concentrated on both sides of the diagonal line, which indicates that the performance of the STiCDRS-NKDE model in wind speed prediction is reliable.

**Figure 13:**
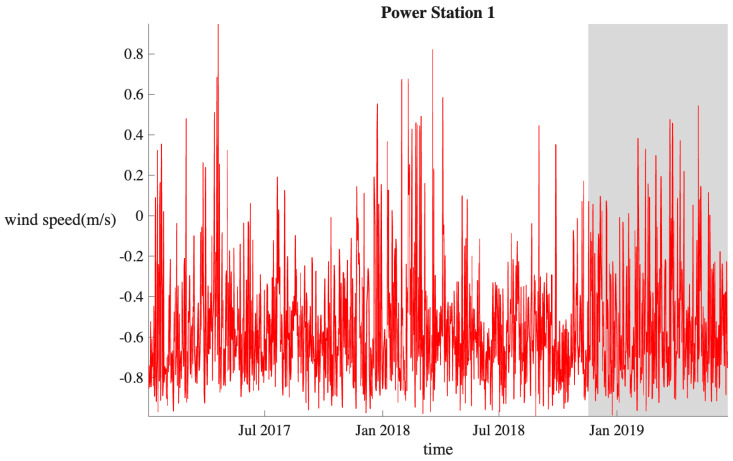
Power Station 1 wind speed dataset on the DataCastle competition platform.

To further demonstrate the effectiveness of the model in predicting wind speed intervals, we utilized the wind speed dataset from the DataCastle competition platform. This dataset includes wind speed data from Power Station 1, measured in meters per second (m/s), with positive values indicating tailwind and negative values indicating headwind. The data is sampled at 15 min intervals, starting from 1 January 2017, at 00:15:00, and ending on 23 June 2019, at 00:00:00, resulting in a total of 86,688 samples. From this, we selected 65,760 samples for the training dataset and 12,489 samples for the testing dataset. The division between the training and testing sets is illustrated in Figure 13.

Figure 13 shows a dataset covering more than two and a half years, exhibiting clear seasonal characteristics, which further validate the model’s performance. It can be observed from the figure that wind speed fluctuations are particularly significant from January to March each year.

As shown in Table 5, with the optimal results highlighted in red, the sub-optimal results highlighted in purple, and the worst results highlighted in green (10 runs were run, with the average being the final result). on the Power Station 1 wind speed dataset, the STiCDRS model achieved significant improvements in multiple key performance indicators compared to other baseline models, particularly in *R*^2^, *MAE*, *RMSE*, and *CP*, demonstrating its superiority and accuracy in wind speed forecasting. Specifically, the positive indicators *R*^2^ and *CP* increased by 0.44% and 0.67%, respectively, while the negative indicators *MAE*, *RMSE*, *MWP*, and *MC* decreased by 12.18%, 10.24%, 4.85%, and 5.49%, respectively. However, due to the more complex structure of the STiCDRS model, training time increased by 18.72%, posing a challenge in the subsequent optimization between the conflicting goals of improving model accuracy and reducing training time.

## 5. Conclusions

In this study, we developed an enhanced deep learning point prediction model, STiCDRS, based on a temporal convolutional network (TCN) that incorporates spatio-temporal feature extraction. By integrating DRSN-CW to optimize the model structure and leveraging the spatio-temporal characteristics of wind speed data, we proposed a hybrid interval prediction framework, STiCDRS-NKDE, which combines deep learning point predictions with the NKDE interval prediction model. Hyper-parameter combinations were adaptively selected using a Bayesian optimization algorithm.

We validated our approach through simulation experiments with four sets of measured wind speed data from Inner Mongolia. Compared with the benchmark model TCN, STiCDRS-NKDE significantly improved interval prediction accuracy and stability. The comprehensive evaluation index MC values were 0.2424, 0.3833, 0.2052, and 0.2070, with average decline rates of 9.04%, 9.15%, 1.20%, and 4.70%, respectively. Additionally, we conducted further validation using the long-term wind speed dataset from DataCastle, achieving promising results. Notably, the performance of STiCDRS-NKDE was the most outstanding among the models evaluated. From comparisons among the six models, we draw the following conclusions:

(1) STiCDRS achieves the highest point prediction accuracy, effectively capturing the nonlinear relationships in the data for more precise predictions.

(2) STiCDRS-NKDE provides the most appropriate prediction confidence intervals, covering the largest number of observation points with the smallest width, and demonstrating greater robustness to noise and uncertainty. The NKDE interval prediction model offers enhanced interpretability of confidence intervals.

(3) STiCDRS-NKDE generates the most reliable probabilistic predictions while maintaining high performance, ensuring optimal confidence interval coverage and effectively capturing the distribution of actual data.

In conclusion, this study demonstrates that the STiCDRS-NKDE model excels in accuracy, uncertainty management, and reliability for short-term wind speed predictions, outperforming other models and providing a robust solution for wind speed forecasting.

## Figures and Tables

**Table 1:** Parameter configuration for all participants in the comparative experiment identification model training.

**CNN/FC Layer Initialization Method**	**Dropout**	**The Order of Each Round of Data**	**Optimizer Methods**	**Loss Function**
Kaiming method	0.5	Random Shuffle	Adam	MSE
**MGM/LSTM layer initialization method**	**Training epochs**	**Training and testing ratio**	**Intermediate layer activation function**	**Number of datasets**
Orthogonal method	100	8:2	ReLU	4

**Table 2:** Bayesian optimization-determined hyper-parameters of TCN model network.

Hyper-Parameter Name	Optimal Value
Convolution kernel size	3
Number of convolution kernels	31
Hidden unit size of fully connected layers	119
Learning rate	0.003
Batch size	64
Regularization coefficient	0.93

**Table 3:** Structure parameters of the proposed STiCDRS framework.

Order	Structural Layer	Structure Parameters	Order	Structural Layer	Structure Parameters
1	Input	8 × 1 × 1	11	Relu-2	31 × 1 × 1
2	DC-conv1	3 × 8 × 31	12	DP-2	3 × 8 × 31
3	BN-1	31 × 1	13	DC-conv4	3 × 8 × 31
4	Relu-1	31 × 1 × 1	14	DRSN-Skip	3 × 8 × 40
5	DP-1	3 × 8 × 31	15	Concatenate-2	3 × 6 × 71
6	DC-conv2	3 × 8 × 31	16	Batch-Normalization-1	71 × 1
7	MGM	8 × 27	17	Full-connect-1	119 × 1
8	Concatenate-1	3 × 8 × 40	18	Dropout-1	119 × 1
9	DC-conv3	3 × 8 × 31	19	Full-connect-2	1
10	BN-2	31 × 1 × 1	20	Point prediction	1

**Table 4:** Evaluation metrics for point and interval predictions across four datasets.

Datasets		Model	CNN	MGM	CLSTM	TCN	STiCDRS	GPR
	Metric							
**dataset1**	***R* ^2^**		0.9901	0.9770	0.9910	0.9883	0.9910	0.9855
	***MAE***		0.3799	0.4950	0.3350	0.3592	0.2995	0.4195
	***RMSE***		0.4909	0.6869	0.4344	0.4884	0.4265	0.5421
	***CP***		0.8125	0.9271	0.8958	0.9271	0.9583	0.9479
	***MWP***		0.2344	0.3235	0.2215	0.2471	0.2323	0.2955
	***MC***		0.2885	0.3490	0.2473	0.2665	0.2424	0.3118
	***ATT***		16.1	33.2	21.4	15.5	16.8	1.9
**dataset2**	***R* ^2^**		0.9958	0.9919	0.9956	0.9957	0.9962	0.9935
	***MAE***		0.2569	0.3507	0.2464	0.2634	0.2500	0.3186
	***RMSE***		0.3461	0.4661	0.3421	0.3623	0.3295	0.4159
	***CP***		0.8815	0.9111	0.8444	0.8519	0.8815	0.9333
	***MWP***		0.3491	0.5179	0.3344	0.3594	0.3378	0.4325
	***MC***		0.3960	0.5685	0.3960	0.4219	0.3833	0.4634
	***ATT***		36.7	45.2	32.4	31.3	33.3	1.7
**dataset3**	***R* ^2^**		0.9924	0.9862	0.9880	0.9918	0.9917	0.9620
	***MAE***		0.1550	0.1909	0.1894	0.1497	0.1585	0.3877
	***RMSE***		0.1956	0.2422	0.2454	0.1891	0.2033	0.4486
	***CP***		1	1	0.9852	1	1	1
	***MWP***		0.2175	0.3149	0.2059	0.2077	0.2052	0.2756
	***MC***		0.2175	0.3149	0.2090	0.2077	0.2052	0.2756
	***ATT***		31.0	41.8	34.4	30.1	32.3	1.8
**dataset4**	***R* ^2^**		0.9957	0.9941	0.9952	0.9964	0.9966	0.9932
	***MAE***		0.3222	0.3344	0.3560	0.2684	0.2570	0.4020
	***RMSE***		0.4075	0.4364	0.4630	0.3443	0.3336	0.5052
	***CP***		0.9583	0.9896	0.8438	0.9948	0.9844	0.9844
	***MWP***		0.2196	0.2676	0.2199	0.2160	0.2038	0.2508
	***MC***		0.2291	0.2704	0.2607	0.2172	0.2070	0.2548
	***ATT***		43.2	48.1	53.3	40.1	41.1	2.1

**Table 5:** Evaluation metrics for point and interval predictions across four datasets.

Datasets		Model	CNN	MGM	CLSTM	TCN	STiCDRS	GPR
	Metric							
**Power Station 1**	***R* ^2^**		0.9531	0.9533	0.9545	0.9577	0.9619	0.9532
	***MAE***		0.4423	0.4541	0.4417	0.3677	0.3229	0.4828
	***RMSE***		0.4577	0.4667	0.4581	0.3722	0.3341	0.5099
	***CP***		0.9122	0.9496	0.9623	0.9766	0.9831	0.9712
	***MWP***		0.2788	0.2931	0.2545	0.2331	0.2218	0.2455
	***MC***		0.3056	0.3087	0.2645	0.2387	0.2256	0.2528
	***ATT***		219.5	288.4	254.1	216.3	256.8	139.8

## Data Availability

The original contributions presented in the study are included in the article, further inquiries can be directed to the corresponding author.
